# Patient Characteristics and Subsequent Health Care Use by Location of SARS-CoV-2 Testing Initiation in a Safety-Net Health System

**DOI:** 10.1001/jamanetworkopen.2021.12857

**Published:** 2021-06-08

**Authors:** Rohan Khazanchi, Tyler N. A. Winkelman, Deepti Pandita, Ryan Jelinek, Riley D. Shearer, Peter J. Bodurtha

**Affiliations:** 1 Health, Homelessness, and Criminal Justice Lab, Hennepin Healthcare Research Institute, Minneapolis, Minnesota; 2 College of Medicine, University of Nebraska Medical Center, Omaha; 3 School of Public Health, University of Minnesota, Minneapolis; 4 Division of General Internal Medicine, Department of Medicine, Hennepin Healthcare, Minneapolis, Minnesota; 5 Division of Clinical Informatics, Hennepin Healthcare, Minneapolis, Minnesota; 6 Division of Hospital Medicine, Hennepin Healthcare, Minneapolis, Minnesota; 7 University of Minnesota Medical School, Minneapolis

## Abstract

This cross-sectional study examines patterns in entry location for SARS-CoV-2 testing in a safety-net health system by patient demographic characteristics.

## Introduction

The COVID-19 pandemic rapidly shifted care delivery toward telehealth and COVID-19 testing services. While utilization differences early in the pandemic have been described among commercially insured patients and in ambulatory care settings,^1,2,3^ health care use patterns for SARS-CoV-2 testing and subsequent health care use remain unclear, especially among safety-net populations.

In this cross-sectional study, we compared a set of patient characteristics across all locations of care initiation for SARS-CoV-2 testing in a safety-net health system in the Minneapolis, Minnesota, area to assess patterns in demographic characteristics and comorbidities.

## Methods

We analyzed electronic health record data from Hennepin Healthcare through November 14, 2020, for SARS-CoV-2 reverse transcription-polymerase chain reaction (RT-PCR) (Roche) and SalivaDirect RT-PCR (Yale School of Public Health) tests among people with viral illness symptoms. We excluded encounters for which testing location could not be ascertained (53 tests [0.1%]).

We classified testing locations from least to most intensive: telehealth, outpatient, emergency department (ED), and inpatient. Telehealth included telephone, video, and asynchronous messaging encounters. Outpatient included clinic visits and community testing events. We defined entry location for SARS-CoV-2 testing as the least intensive encounter within 5 days prior to test collection to account for delays between telehealth visits and testing.

We compared patient sociodemographic and clinical characteristics between locations. Patient race and ethnicity were defined based on self-reported identities noted in the electronic health record (ie, Hispanic and non-Hispanic White, Black, Native American, and Asian or Pacific Islander) and recoded as mutually exclusive categories, with Hispanic patients first labeled as Hispanic and remaining patients designated according to race. Comorbidities were identified using *International Statistical Classification of Diseases, Tenth Revision, Clinical Modification* (*ICD-10-CM*) diagnosis codes (eTable in the Supplement) from December 27, 2015, through the end of the study period. We also examined relationships between entry location and the most intensive encounter occurring within 21 days following test collection.

We performed descriptive analyses using R version 4.0.2 (R Project for Statistical Computing). Hennepin Healthcare Research Institute’s institutional review board approved this study, and granted an exemption for informed consent requirements based on the use of deidentified data, in accordance with 45 CFR 46.116. We followed the Strengthening the Reporting of Observational Studies in Epidemiology (STROBE) reporting guideline for cross-sectional studies.

## Results

We identified 67 317 tests among patients with symptoms (median [interquartile range] age, 33 [24-48] years; 35 292 [52.4%] women; 26 595 [39.5%] White, 12 596 [18.7%] Black, and 3276 [4.9%] Asian patients); 58 561 tests (87.0%) had an outpatient testing entry location. SARS-CoV-2 positivity rates varied by entry location, ranging from 9.5% to 22.9%.

Patient demographic characteristics and comorbidities differed by entry location (Table 1). Black patients accounted for 9.0% (467 tests) of telehealth-initiated tests and 45.1% (898) of ED-initiated tests, compared with 64.5% (3356 tests) and 23.7% (473 tests), respectively, for White patients. Interpreter services were used over 6 times more often for outpatient-initiated tests compared with telehealth (14.5% [8517 tests] vs 2.3% [122 tests]). Patients with 1 or more comorbidities accounted for a greater share of tests initiated through the ED (44.6% [888 tests]) than outpatient (14.9% [8709 tests]) or telehealth (20.2% [1052 tests]).

**Table 1.** Characteristics of Study Population by Location of Initial Presentation for Symptomatic SARS-CoV-2 Testing

Characteristic^a^	Symptomatic SARS-CoV-2 tests by location of initial presentation, No. (%)				
	All locations (N = 67 317)	Telehealth (n = 5207)	Outpatient (n = 58 561)	Emergency department (n = 1992)	Inpatient (n = 1504)
Positive SARS-CoV-2 tests	10 863 (16.1)	494 (9.5)	9612 (16.4)	456 (22.9)	297 (19.7)
Age, median (IQR), y	33 (24-48)	33 (26-42)	33 (23-48)	37 (26-51)	57 (37-68)
0-24	17 822 (26.5)	935 (18.0)	16 333 (27.9)	403 (20.2)	138 (9.2)
25-49	33 904 (50.4)	3476 (66.8)	28 935 (49.4)	1030 (51.7)	442 (29.4)
50-64	10 850 (16.1)	648 (12.4)	9357 (16.0)	390 (19.6)	440 (29.3)
≥65	4741 (7.0)	148 (2.8)	3936 (6.7)	169 (8.5)	484 (32.2)
Gender					
Female	35 292 (52.4)	3149 (60.5)	30 589 (52.2)	922 (46.3)	606 (40.3)
Male	32 019 (47.5)	2057 (39.5)	27 967 (47.8)	1070 (59.7)	898 (59.7)
Race/ethnicity^b^					
Non-Hispanic					
White	26 595 (39.5)	3356 (64.5)	22 178 (37.9)	473 (23.7)	566 (37.6)
Black or African American	12 596 (18.7)	467 (9.0)	10 681 (18.2)	898 (45.1)	537 (35.7)
Native American	861 (1.3)	29 (0.6)	642 (1.1)	107 (5.4)	80 (5.4)
Asian or Pacific Islander	3276 (4.9)	298 (5.7)	2889 (4.9)	40 (2.0)	48 (2.0)
Multiracial	861 (1.3)	62 (1.2)	733 (1.3)	43 (2.2)	22 (1.5)
Hispanic	11 096 (16.5)	387 (7.4)	10 124 (17.3)	382 (19.2)	193 (12.8)
Unknown	12 032 (17.9)	608 (11.7)	11 314 (19.3)	49 (2.5)	58 (3.9)
Preferred language					
English	46 901 (69.7)	4589 (88.1)	39 541 (67.5)	1556 (78.1)	1175 (78.1)
Spanish	7488 (11.1)	97 (1.9)	6984 (11.9)	250 (12.6)	151 (10.0)
Somali	1493 (2.2)	NR^c^	1252 (2.1)	134 (6.7)	89 (5.9)
Other non-English	1149 (1.7)	24 (0.4)	1010 (1.7)	48 (2.4)	66 (1.9)
Unknown	10 286 (15.3)	482 (9.3)	9774 (16.7)	NR^c^	23 (5.7)
Use of interpreter services	9340 (13.9)	122 (2.3)	8517 (14.5)	395 (19.8)	296 (19.7)
BMI, median (IQR)	27.33 (23.1-32.3)	27.26 (23.0-32.3)	27.31 (23.1-32.167	27.2 (22.9-32.3)	27.92 (24.0-34.2)
No. of comorbidities					
None	55 627 (82.6)	4155 (79.8)	49 852 (85.1)	1104 (55.4)	487 (32.4)
1	6042 (9.0)	649 (12.5)	4683 (8.0)	428 (21.5)	273 (18.2)
≥2	5648 (8.4)	403 (7.7)	4026 (6.9)	460 (23.1)	744 (49.5)
Comorbid conditions					
Asthma	4075 (6.1)	484 (9.3)	2936 (5.0)	403 (20.2)	252 (16.8)
Cancer	717 (1.1)	48 (0.9)	548 (5.0)	27 (1.4)	94 (6.3)
Chronic kidney disease	1400 (2.1)	79 (1.5)	944 (0.9)	93 (4.7)	284 (18.9)
Chronic obstructive pulmonary disease	2384 (3.6)	191 (3.7)	1637 (1.6)	246 (12.3)	310 (20.6)
Diabetes	3873 (5.8)	233 (4.5)	2907 (2.8)	252 (12.7)	481 (32.0)
Cardiovascular or cerebrovascular disease	2857 (4.3)	162 (3.1)	1895 (3.2)	245 (12.3)	555 (36.9)
HIV	617 (0.9)	77 (1.5)	460 (0.8)	47 (2.4)	33 (2.2)
Hypertension	6543 (9.7)	495 (9.5)	4823 (8.2)	476 (23.9)	749 (49.8)
Substance use disorders					
Alcohol	3594 (5.4)	266 (5.1)	2527 (4.3)	422 (21.2)	379 (25.2)
Cocaine	988 (1.5)	68 (1.3)	653 (1.1)	144 (7.2)	123 (8.2)
Methamphetamine	1421 (2.1)	98 (1.9)	940 (1.6)	191 (9.6)	192 (12.8)
Opioids	1290 (1.9)	87 (1.7)	899 (1.5)	172 (8.6)	132 (8.8)

Abbreviations: BMI, body mass index (calculated as weight in kilograms divided by height in meters squared); IQR, interquartile range; NR, not reported; SARS-CoV-2, severe acute respiratory syndrome coronavirus 2.

^a^ Rows indicating the number of individuals with missing data for a given measure were redacted if fewer than 20 individuals in any given cell had missing data. Thus, column and row totals for each measure may not sum to column or row totals.

^b^ Race and ethnicity definitions are mutually exclusive and reflect self-reported identities collected from the electronic health record. Hispanic patients were first labeled as Hispanic, then remaining patients were designated according to race. Multiracial reflects non-Hispanic patients with multiple race designations. Unknown race or ethnicity represents patients with missing race and missing Hispanic ethnicity data.

^c^ Values censored for data privacy (<20 patients).

Maximum level of care also varied between testing entry locations (Table 2). Among patients who initiated testing via the ED, 154 (7.7%) were hospitalized, and 500 (33.2%) patients initially tested as inpatients were admitted to the intensive care unit.

**Table 2.** Location of Initial Presentation for Symptomatic SARS-CoV-2 Testing by Maximum Level of Care

Maximum level of care	Symptomatic SARS-CoV-2 tests by location of initial presentation, No. (%)				
	All locations (n = 67 317)	Telehealth (n = 5207)	Outpatient (n = 58 561)	ED (n = 1992)	Inpatient (n = 1504)
Outpatient	61 646 (91.6)	4983 (95.7)	56 660 (96.8)	NR^a^	NR^a^
ED, not admitted	3081 (4.6)	135 (2.6)	1124 (1.1)	1808 (90.8)	NR^a^
Non-ICU hospital admission	1893 (2.8)	74 (1.4)	658 (1.1)	154 (7.7)	1004 (66.8)
ICU admission	659 (0.1)	NR^a^	116 (0.2)	29 (1.5)	500 (33.2)

Abbreviations: ED, emergency department; ICU, intensive care unit; NR, not reported; SARS-CoV-2, severe acute respiratory syndrome coronavirus 2.

^a^ Values censored for data privacy (<20 patients).

## Discussion

We found differences in sociodemographic and clinical characteristics by entry location for SARS-CoV-2 testing within our safety-net health system. White and English-speaking individuals disproportionately initiated testing via telehealth visits, while Black, Native American, and non–English-speaking patients disproportionately initiated testing through the ED. These racial/ethnic and language inequities in entry location intensity may be explained by structural barriers to timely testing access, delayed care seeking, and increased comorbidity burden among patients with acute presentations, as well as clinician- and practice-level variation in telehealth use.^2,3,4,5,6^

Testing initiated via telehealth and outpatient encounters was associated with lower rates of subsequent inpatient and intensive care unit care than testing initiated in more intensive settings. Although these are expected findings, health systems could leverage these associations between testing location and acuity to anticipate hospitalization surges.

A key limitation to this study is that our single-system analysis cannot capture service use in other systems. Nonetheless, our findings highlight demographic and clinical differences in health care use for SARS-CoV-2 testing across all care delivery settings, which can strategically inform outreach efforts for distinct populations. Without structural reforms, rapid implementation of telehealth and other new services may exacerbate inequities in access to care,^2,3,4,5^ particularly if these investments come at the expense of other care sites.

## Notes

Supplement. **eTable.** Reference Codes for Comorbidities of Interest From *International Classification of Disease, 10th Revision, Clinical Modification* (*ICD-10-CM*)
