# Supplementary material for: Patient Characteristics and Subsequent Health Care Use by Location of SARS-CoV-2 Testing Initiation in a Safety-Net Health System
Source: JAMA Netw Open. 2021 Jun 8;4(6):e2112857. doi: 10.1001/jamanetworkopen.2021.12857 (PMC8188267; doi:10.1001/jamanetworkopen.2021.12857)
Supplement: Supplement. — eTable. Reference Codes for Comorbidities of Interest From International Classification of Disease, 10th Revision, Clinical Modification (ICD-10-CM) [file jamanetwopen-e2112857-s001.pdf]

Supplemental Online Content

Khazanchi R, Winkelman TNA, Pandita D, Jelinek R, Shearer RD, Bodurtha PJ. Patient characteristics and subsequent health care use by location of SARS-CoV-2 testing initiation in a safety-net health system. *JAMA Netw Open.* 2021;4(6):e2112857. doi:10.1001/jamanetworkopen.2021.12857

eTable. Reference Codes for Comorbidities of Interest From *International Classification of Disease, 10th Revision, Clinical Modification* (*ICD-10-CM*)

This supplemental material has been provided by the authors to give readers additional information about their work.

© 2021 Khazanchi R et al. *JAMA Network Open.*

eTable. Reference codes for comorbidities of interest from *International Classification of Disease, 10th revision, Clinical Modification (ICD-10-CM)*

**Comorbidity**	**ICD-10 Codes**
Asthma	J45.20, J45.21, J45.22, J45.30, J45.31, J45.32, J45.40, J45.41, J45.42, J45.50, J45.51, J45.52, J45.901, J45.902, J45.909, J45.990, J45.991, J45.998
Cancer	Breast Cancer<br>C50.011, C50.012, C50.019, C50.021, C50.022, C50.029, C50.111, C50.112, C50.119, C50.121, C50.122, C50.129, C50.211, C50.212, C50.219, C50.221, C50.222, C50.229, C50.311, C50.312, C50.319, C50.321, C50.322, C50.329, C50.411, C50.412, C50.419, C40.421, C50.422, C50.429, C50.511, C50.512, C50.519, C50.521, C50.522, C50.529, C50.611, C50.612, C50.619, C50.621, C50.622, C50.629, C50.811, C50.812, C50.819, C50.821, C50.822, C50.829, C50.911, C50.912, C50.919, C50.921, C50.922, C50.929, D05.00, D05.01, D05.02, D05.10, D05.11, D05.12, D05.80, D05.81, D05.82, D05.90, D05.91, D05.92, Z85.3<br><br>Colorectal Cancer<br>C18.0, C18.1, C18.2, C18.3, C18.4, C18.5, C18.6, C18.7, C18.8, C18.9, C19, C20, D01.0, D01.1, D01.2, Z85.038, Z85.048<br><br>Endometrial Cancer<br>C54.1, C54.2, C54.3, C54.9, D07.0, Z85.42<br><br>Leukemia or Lymphoma<br>C81.00, C81.01, C81.02, C81.03, C81.04, C81.05, C81.06, C81.07, C81.08, C81.09, C81.10, C81.11, C81.12, C81.13, C81.14, C81.15, C81.16, C81.17, C81.18, C81.19, C81.20, C81.21, C81.22, C82.23, C81.24, C81.25, C81.26, C81.27, C81.28, C81.29, C81.30, C81.31, C81.32, C82.33, C81.34, C81.35, C81.36, C81.37, C81.38, C81.39, C81.40, C81.41, C81.42, C82.43, C81.44, C81.45, C81.46, C81.47, C81.48, C81.49, C81.70, C81.71, C81.72, C82.73, C81.74, C81.75, C81.76, C81.77, C81.78, C81.79, C81.90, C81.91, C81.92, C82.93, C81.94, C81.95, C81.96, C81.97, C81.98, C81.99, C82.00, C82.01, C82.02, C82.03, C82.04, C82.05, C82.06, C82.07, C82.08, C82.09, C82.10, C82.11, C82.12, C82.13, C82.14, C82.15, C82.16, C82.17, C82.18, C82.19, C82.20, C82.21, C82.22, C82.23, C82.24, C82.25, C82.26, C82.27, C82.28, C82.29, C82.30, C82.31, C82.32, C82.33, C82.34, C82.35, C82.36, C82.37, C82.38, C82.39, C82.40, C82.41, C82.42, C82.43, C82.44, C82.45, C82.46, C82.47, C82.48, C82.49, C82.50, C82.51, C82.52, C82.53, C82.54, C82.55, C82.56, C82.57, C82.58, C82.59, C82.60, C82.61, C82.62, C82.63, C82.64, C82.65, C82.66, C82.67, C82.68, C82.69, C82.80, C82.81, C82.82, C82.83, C82.84, C82.85, C82.86, C82.87, C82.88, C82.89, C82.90, C82.91, C82.92, C82.93, C82.94, C82.95, C82.96, C82.97, C82.98, C82.99, C83.00, C83.01, C83.02, C83.03, C83.04, C83.05, C83.06, C83.07,

© 2021 Khazanchi R et al. *JAMA Network Open.*

	C83.08, C83.09, C83.10, C83.11, C83.12, C83.13, C83.14, C83.15, C83.16, C83.17, C83.18, C83.19, C83.30, C83.31, C83.32, C83.33, C83.34, C83.35, C83.36, C83.37, C83.38, C83.39, C83.50, C83.51, C83.52, C83.53, C83.54, C83.55, C83.56, C83.57, C83.58, C83.59, C83.70, C83.71, C83.72, C83.73, C83.74, C83.75, C83.76, C83.77, C83.78, C83.79, C83.80, C83.81, C83.82, C83.83, C83.84, C83.85, C83.86, C83.87, C83.88, C83.89, C83.90, C83.91, C83.92, C83.93, C83.94, C83.95, C83.96, C83.97 C83.98, C83.99, C84.00, C84.01, C84.02, C84.03, C84.04, C84.05, C84.06, C84.07, C84.08, C84.09, C84.10, C84.11, C84.12, C84.13, C84.14, C84.15, C84.16, C84.17, C84.18, C84.19, C84.40, C84.41, C84.42, C84.43, C84.44, C84.45, C84.46, C84.47, C84.48, C84.49, C84.60, C84.61, C84.62, C84.63, C84.64, C84.65, C84.66, C84.67, C84.68, C84.69, C84.70, C84.71, C84.72, C84.73, C84.74, C84.75, C84.76, C84.77, C84.78, C84.79, C84.90, C84.91, C84.92, C84.93, C84.94, C84.95, C84.96, C84.97, C84.98, C84.99, C84.A0, C84.A1, C84.A2, C84.A3, C84.A4, C84.A5, C84.A6, C84.A7, C84.A8, C84.A9, C84.Z0, C84.Z1, C84.Z2, C84.Z3, C84.Z4, C84.Z5, C84.Z6, C84.Z7, C84.Z8, C84.Z9, C85.10, C85.11, C85.12, C85.13, C85.14, C85.15, C85.16, C85.17, C85.18, C85.19, C85.20, C85.21, C85.22, C85.23, C85.24, C85.25, C85.26, C85.27, C85.28, C85.29, C85.80, C85.81, C85.82, C85.83, C85.84, C85.85, C85.86, C85.87, C85.88, C85.89, C85.90, C85.91, C85.92, C85.93, C85.94, C85.95, C85.96, C85.97, C85.98, C85.99, C86.0, C86.1, C86.2, C86.3, C86.4, C86.5, C86.6, C88.4, C90.10, C90.11, C90.12, C91.00, C91.01, C91.02, C91.10, C91.11, C91.12, C91.30, C91.31, C91.32, C91.40, C91.41, C91.42, C91.50, C91.51, C91.52, C91.60, C91.61, C91.62, C91.A0, C91.A1, C91.A2, C91.Z0, C91.Z1, C91.Z2, C91.90, C91.91, C91.92, C92.00, C92.01, C92.02, C92.10, C92.11, C92.12, C92.20, C92.21, C92.22, C92.30, C92.31, C92.32, C92.40, C92.41, C92.42, C92.50, C92.51, C92.52, C92.60, C92.61, C92.62, C92.90, C92.91, C92.92, C92.A0, C92.A1, C92.A2, C92.Z0, C92.Z1, C92.Z2, C93.00, C93.01, C93.02, C93.10, C93.11, C93.12, C93.30, C93.31, C93.32, C93.Z0, C93.Z1, C93.Z2, C93.90, C93.91, C93.92, C94.00, C94.01, C94.02, C94.20, C94.21, C94.22, C94.30, C94.31, C94.32, C94.80, C94.81, C94.82, C95.00, C95.01, C95.02, C95.10, C95.11, C95.12, C95.90, C95.91, C95.92, C96.4, C96.9, C96.Z, D45, Z85.6, Z85.71, Z85.72, Z85.79<br><br>Lung Cancer<br>C34.00, C34.01, C34.02, C34.10, C34.11, C34.12, C34.2, C34.30, C34.31, C34.32, C34.80, C34.81, C34.82, C34.90, C34.91, C34.92, D02.20, D02.21, D02.22, Z85.118<br><br>Prostate Cancer<br>C61, D07.5, Z85.46
Chronic kidney disease	N18.1, N18.2, N18.3, N18.4M N18.5, N18.9, E08.22, E09.22, E10.22, E11.22, E13.22, I12.0, I12.9, I13.0, I13.2

© 2021 Khazanchi R et al. *JAMA Network Open.*

Chronic obstructive pulmonary disease (COPD)	J40, J41.0, J41.1, J41.8, J42, J43.0, J43.1, J43.2, J43.8, J43.9, J44.0, J44.1, J44.9, J47.0, J47.1, J47.9
Diabetes mellitus	E08.00, E08.01, E08.10, E08.11, E08.21, E08.22, E08.29, E08.311, E08.319, E08.321, E08.329, E08.331, E08.339, E08.341, E08.349, E08.351, E08.359, E08.36, E08.39, E08.40, E08.41, E08.42, E08.43, E08.44, E08.49, E08.51, E08.52, E08.59, E08.610, E08.618, E08.620, E08.621, E08.622, E08.628, E08.630, E08.638, E08.641, E08.649, E08.65, E08.69, E08.8, E08.9, E09.00, E09.01, E09.10, E09.11, E09.21, E09.22, E09.29, E09.311, E09.319, E09.321, E09.329, E09.331, E09.339, E09.341, E09.349, E09.351, E09.359, E09.36, E09.39, E09.40, E09.41, E09.42, E09.43, E09.44, E09.49, E09.51, E09.52, E09.59, E09.610, E09.618, E09.620, E09.621, E09.622, E09.628, E09.630, E09.638, E09.641, E09.649, E09.65, E09.69, E09.8, E09.9, E10.10, E10.11, E10.21, E10.22, E10.29, E10.311, E10.319, E10.321, E10.329, E10.331, E10.339, E10.341, E10.349, E10.351, E10.359, E10.36, E10.39, E10.40, E10.41, E10.42, E10.43, E10.44, E10.49, E10.51, E10.52, E10.59, E10.610, E10.618, E10.620, E10.621, E10.622, E10.628, E10.630, E10.638, E10.641, E10.649, E10.65, E10.69, E10.8, E10.9, E11.00, E11.01, E11.21, E11.22, E11.29, E11.311, E11.319, E11.321, E11.329, E11.331, E11.339, E11.341, E11.349, E11.351, E11.359, E11.36, E11.39, E11.40, E11.41, E11.42, E11.43, E11.44, E11.49, E11.51, E11.52, E11.59, E11.610, E11.618, E11.620, E11.621, E11.622, E11.628, E11.630, E11.638, E11.641, E11.649, E11.65, E11.69, E11.8, E11.9, E13.00, E13.01, E13.10, E13.11, E13.21, E13.22, E13.29, E13.311, E13.319, E13.321, E13.329, E13.331, E13.339, E13.341, E13.349, E13.351, E13.359, E13.36, E13.39, E13.40, E13.41, E13.42, E13.43, E13.44, E13.49, E13.51, E13.52, E13.59, E13.610, E13.618, E13.620, E13.621, E13.622, E13.628, E13.630, E13.638, E13.641, E13.649, E13.65, E13.69, E13.8, E13.9
Cardiovascular/Cerebrovascular disease	Acute Myocardial Infarction<br>I21.01, I21.02, I21.09, I21.11, I21.19, I21.21, I21.29, I21.3, I21.4, I22.0, I22.1, I22.2, I22.8, I22.9<br><br>Heart Failure<br>I09.81, I11.0, I13.0, I13.2, I50.1, I50.20, I50.21, I50.22, I50.23, I50.30, I50.31, I50.32, I50.33, I50.40. I50.41, I50.42, I50.43, I50.9<br><br>Ischemic Heart Disease<br>I20.0, I20.1, I20.8, I20.9, I21.01, I21.02, I21.09, I21.11, I21.19, I21.21, I21.29, I21.3, I21.4, I22.0, I22.1, I22.2, I22.8, I22.9, I24.0, I24.1, I24.8, I24.9, I25.10, I25.110, I25.111, I25.118, I25.119, I25.2, I25.42, I25.5, I25.6, I25.700, I25.701, I25.708, I25.709, I25.710, I25.711, I25.718, I25.719, I25.720, I25.721, I25.728, I25.729, I25.730, I25.731, I25.738, I25.739, I25.750, I25.751, I25.758, I25.759, I25.760, I26.761, I25.768, I25.769, I25.790, I25.791, I25.798, I25.799, I25.810, I25.811, I25.812, I25.82, I25.83, I25.84, I25.89, I25.9

	Peripheral Vascular Disease<br>E08.51, E08.52, E09.51, E09.52, E10.51, E10.52, E11.51, E11.52, E13.51, E13.52, I70.0, I70.1, I70.201, I70.202, I70.203, I70.208, I70.209, I70.211, I70.212, I70.213, I70.218, I70.219, I70.221, I70.222, I70.223, I70.228, I70.229, I70.231, I70.232, I70.233, I70.234, I70.235, I70.238, I70.239, I70.241, I70.242, I70.243, I70.244, I70.245, I70.248, I70.249, I70.25, I70.291, I70.292, I70.293, I70.298, I70.299, I70.92, I73.81, I73.89, I73.9, I79.1, I79.8<br><br>Stroke or Transient Ischemic Attack<br>G45.0, G45.1, G45.2, G45.8, G45.9, G46.0, G46.1, G46.2, G97.31, G97.32, I60.00, I60.01, I60.02, I60.10, I60.11, I60.12, I60.20, I60.21, I60.22, I60.30, I60.31, I60.32, I60.4, I60.50, I60.51, I60.52, I60.6, I60.7, I60.8, I60.9, I61.0, I61.1, I61.2, I61.3, I61.4, I61.5, I61.6, I61.8, I61.9, I63.00, I63.02, I63.011, I63.012, I63.019, I63.031, I63.032, I63.039, I63.09, I63.10, I63.111, I63.112, I63.119, I63.12, I63.131, I63.132, I63.139, I63.19, I63.20, I63.211, I63.212, I63.219, I63.22, I63.231, I63.232, I63.239, I63.29, I63.30, I63.311, I63.312, I63.319, I63.321, I63.322, I63.329, I63.331, I63.332, I63.339, I63.341, I63.342, I63.349, I63.39, I63.40, I63.411, I63.412, I63.419, I63.421, I63.422, I63.429, I63.431, I63.432, I63.439, I63.441, I63.442, I63.449, I63.49, I63.50, I63.511, I63.512, I63.519, I63.521, I63.522, I63.529, I63.531, I63.532, I63.539, I63.541, I63.542, I63.549, I63.59, I63.6, I63.8, I63.9, I66.01, I66.02, I66.03, I66.09, I66.11, I66.12, I66.13, I66.19, I66.21, I66.22, I66.23, I66.29, I66.3, I66.8, I66.9, I67.841, I67.848, I67.89, I97.810, I97.811, I97.820, I97.821
HIV	B20, B97.35, R75, Z21
Hypertension	H35.031, H35.032, H35.033, H35.039, I10, I11.0, I11.9, I12.0, I12.9, I13.0, I13.10, I13.11, I13.2, I15.0, I15.1, I15.2, I15.8, I15.9, I67.4, N26.2
Alcohol use disorder	F10.10, F10.120, F10.121, F10.129, F10.14, F10.150, F10.151, F10.159, F10.180, F10.181, F10.182, F10.188, F10.19, F10.20, F10.220, F10.221, F10.229, F10.230, F10.231, F10.232, F10.239, F10.24, F10.250, F10.251, F10.259, F10.26, F10.27, F10.280, F10.281, F10.282, F10.288, F10.29, F10.920, F10.921, F10.929, F10.94, F10.950, F10.951, F10.959, F10.96, F10.97, F10.980, F10.981, F10.982, F10.988, F10.99, G62.1, I42.6, K29.20, K29.21, K70.0, K70.10, K70.11, K70.2, K70.30, K70.31, K70.40, K70.41, K70.9, O35.4XX0, O35.4XX1, O35.4XX2, O35.4XX3, O35.4XX4, O35.4XX5, O35.4XX9, O99.310, O99.311, O99.312, O99.313, O99.314, O99.315
Cocaine use disorder	F14.10, F14.120, F14.121, F14.122, F14.129, F14.14, F14.150, F14.151, F14.159, F14.180, F14.181, F14.182, F14.188, F14.19, F14.20, F14.220, F14.221, F14.222, F14.229, F14.23, F14.24, F14.250, F14.251, F14.259, F14.280, F14.281, F14.282, F14.288, F14.29, F14.90, F14.920, F14.921, F14.922, F14.929, F14.94,

© 2021 Khazanchi R et al. *JAMA Network Open.*

	F14.950, F14.951, F14.959, F14.980, F14.981, F14.982, F14.988, F14.99, T40.5X1A, T40.5X3A, T40.5X4A, T40.5X5A
Methamphetamines use disorder	F15.10, F15.120, F15.121, F15.122, F15.129, F15.14, F15.150, F15.151, F15.159, F15.180, F15.181, F15.182, F15.188, F15.19, F15.20, F15.220, F15.221, F15.222, F15.229, F15.23, F15.24, F15.250, F15.251, F15.259, F15.280, F15.281, F15.282, F15.288, F15.29, F15.90, F15.920, F15.921, F15.922, F15.929, F15.93, F15.94, F15.950, F15.951, F15.959, F15.980, F15.981, F15.982, F15.988, F15.99, T43.601A, T43.601D, T43.601S, T43.603A, T43.603D, T43.603S, T43.604A, T43.604D, T43.604S, T43.605A, T43.605D, T43.605S, T43.621A, T43.621D, T43.621S, T43.622A, T43.622D, T43.622S, T43.623A, T43.623D, T43.623S, T43.624A, T43.624D, T43.624S, T43.625A, T43.625D, T43.625S, T43.641A, T43.641D, T43.641S, T43.642A, T43.642D, T43.642S, T43.643A, T43.643D, T43.643S, T43.644A, T43.644D, T43.644S, T43.691A, T43.691D, T43.691S, T43.692A, T43.692D, T43.692S, T43.693A, T43.693D, T43.693S, T43.694A, T43.694D, T43.694S, T43.695A, T43.695D, T43.695S
Opioid use disorder	F11.10, F11.120, F11.121, F11.122, F11.129, F11.14, F11.150, F11.151, F11.159, F11.181, F11.182, F11.188, F11.19, F11.20, F11.220, F11.221, F11.222, F11.229, F11.23, F11.24, F11.250, F11.251, F11.259, F11.281, F11.282, F11.288, F11.29, F11.90, F11.920, F11.921, F11.922, F11.929, F11.93, F11.94, F11.950, F11.951, F11.959, F11.981, F11.982, F11.988, F11.99, T40.0X1A, T40.0X3A, T40.0X4A, T40.0X5A, T40.1X1A, T40.1X3A, T40.1X4A, T40.2X1A, T40.2X3A, T40.2X4A, T40.2X5A, T40.3X1A, T40.3X3A, T40.3X4A, T40.3X5A, T40.4X1A, T40.4X3A, T40.4X4A, T40.4X5A, T40.601A, T40.603A, T40.604A, T40.605A, T40.691A, T40.693A, T40.694A, T40.695A

© 2021 Khazanchi R et al. *JAMA Network Open.*
